# Exploring athletic expertise and conflict processing: behavioral and neural responses to head fakes and flanker tasks

**DOI:** 10.3389/fnins.2025.1519011

**Published:** 2025-03-12

**Authors:** Siyu Gao, Zhibo Sun, Danlei Wang, Arash Mirifar, Chenglin Zhou, Mengkai Luan

**Affiliations:** ^1^School of Psychology, Shanghai University of Sport, Shanghai, China; ^2^School of Athletic Performance, Shanghai University of Sport, Shanghai, China; ^3^Center for the Study of Emotion and Attention, University of Florida, Gainesville, FL, United States; ^4^Research Center for Exercise and Brain Science, Shanghai University of Sport, Shanghai, China; ^5^Key Laboratory of Sports Cognition Assessment and Regulation of the General Administration of Sport of China, Shanghai University of Sport, Shanghai, China

**Keywords:** head fake, deceptive action, kinematic information, social cue, conflict processing, ERP

## Abstract

Deceptive actions in sports, such as head fakes, present cognitive challenges by misleading opponents with irrelevant cues, requiring individuals to resolve conflicting information. This study investigates how athletic expertise influences the processing of deceptive actions and broader conflict scenarios by comparing the behavioral and neural responses of basketball players and non-athletes across three tasks: the head-fake task, the flanker task, and the face-viewpoint direction flanker task. The behavioral results revealed that athletes exhibited shorter reaction times in the head-fake and face-viewpoint direction flanker tasks compared to non-athletes, suggesting an expertise advantage in processing kinematic information and social cues, while no group differences were observed in the flanker task involving non-social stimuli. ERP findings in the head-fake task revealed that non-athletes exhibited larger amplitudes in an early negative component over fronto-central electrodes and an early positive component over parieto-occipital electrodes compared to athletes, regardless of congruency, indicating different neural engagement between the groups. Further analysis suggests that these components may reflect a shared neural process for the entire action processing, rather than distinct processes for conflict resolution. Across all tasks, a significant congruency effect was observed, with faster and more accurate responses in congruent conditions compared to incongruent ones. However, no group-by-congruency interaction effects were found, indicating that athletic expertise does not provide a general advantage in conflict processing. Overall, our findings suggest that athletic expertise enhances the processing of kinematic and social information, but does not confer an advantage in conflict processing.

## Introduction

Understanding others’ intentions is a vital element of social interactions, where individuals often rely on nonverbal cues to judge whether someone is being honest or deceptive. On this point, interpreting bodily movements plays a critical role in identifying potential deceit (Tidoni et al., 2013; Vrij, 2004). In the context of competitive sports, this skill is similarly crucial, as athletes frequently encounter situations where they must quickly interpret an opponent’s intentions (Williams and Jackson, 2019). However, in many cases, opponents purposely provide misleading signals, a tactic known as deceptive action (Jackson et al., 2006; Ripoll et al., 1995). These actions create cognitive conflict by presenting conflicting information about the opponent’s true intentions, forcing the athlete to resolve the conflict and make rapid decisions. Consequently, athletes may be misled into responding to false cues, thus giving the opponent who executed the deceptive action an advantage (Güldenpenning et al., 2017; Wright et al., 2013; Wright and Jackson, 2014).

A prominent example of such deception is the head fake in basketball, where an athlete is misled by the irrelevant cue of their opponent’s head direction, which suggests a movement in one direction while the actual action—such as a pass—occurs in the opposite, relevant direction (Güldenpenning et al., 2020; Kunde et al., 2011; Weigelt et al., 2017). In this scenario, the athlete must quickly distinguish between the deceptive, irrelevant information (head direction) and the relevant action (pass direction) to respond correctly. This situation exemplifies what is known as the head fake effect, where the conflict between the misleading head movement and the actual action creates a delay in the opponent’s response time or leads to errors in judgment (e.g., Güldenpenning et al., 2022, 2024; Kunde et al., 2011). By diverting attention from the relevant cue—the ball’s trajectory—the deceptive head movement creates cognitive interference, making it harder for the athlete to focus on critical information (Kunde et al., 2011).

Numerous studies using the expert-novice approach have shown that expert athletes outperform novices in recognizing and responding to deceptive movements across various sports. For instance, experts are better at identifying deceptive changes in running direction in rugby (Jackson et al., 2006) and distinguishing between real and fake throws in team handball (Cañal-Bruland and Schmidt, 2009). This advantage is often attributed to experts’ superior ability to process kinematic information—such as body posture, motion trajectories, and speed—which allows them to better interpret opponents’ movements and respond effectively (Aglioti et al., 2008; Williams and Jackson, 2019). However, when it comes to head fakes specifically, the evidence remains inconclusive. For example, Weigelt et al. (2017) found that expert basketball players did not show a reduced head-fake effect compared to non-athletes. In contrast, Güldenpenning et al. (2020) found that participants exposed to dynamic head-fake stimuli over multiple sessions showed a significant reduction in the head-fake effect. Similarly, Güldenpenning et al. (2022) reported that basketball players exhibited a smaller head-fake effect compared to the novice participants. Moreover, the neural mechanisms underlying deceptive actions in sports have received limited attention. As a result, it remains unclear whether and how athletic expertise influences the processing and response to head fakes.

Even if athletes demonstrate an advantage in responding to head fakes, it is unclear whether this advantage is specific to the demands of their particular sport or reflects a broader ability to handle conflicting information across various contexts. Athletes in open-skill sports (e.g., basketball, soccer) frequently operate in dynamic, unpredictable environments that require them to resolve conflicts and make rapid decisions (Wang et al., 2017; Wang C.-H. et al., 2020; Wang L. et al., 2020; Voss et al., 2010). This could suggest that their expertise in conflict processing extends beyond domain-specific challenges like head fakes to more general forms of cognitive control. Alternatively, the expertise may not be universally applicable to all types of conflict but could instead reflect an enhanced ability to process social cues, such as head direction and gaze, which are critical in both sports and social interactions (Ji et al., 2020, 2022; King et al., 2012; Schütz et al., 2020). Thus, athletes may develop a heightened capacity to ignore misleading gaze direction or head movements, an ability that could apply beyond sports to managing conflicting social signals more broadly.

To explore whether athletic expertise affects the ability to process deceptive actions and broader conflict scenarios, we examined the differences between basketball players and a control group across three tasks: the head-fake task, the flanker task, and the face-viewpoint direction flanker task. These tasks were selected to assess both sport-specific (head fakes) and more general (the flanker task and the face-viewpoint direction flanker task) cognitive conflict control. In addition to behavioral performance (reaction time and accuracy), electroencephalography (EEG) data were simultaneously recorded to provide insights into the neural mechanisms underlying conflict processing in athletes and non-athletes. For the flanker tasks (including both the arrow-based and face-viewpoint direction versions), we focused on the N2 and P3 components, which are well-documented in the literature for reflecting cognitive control and evaluative processing. The N2 component is typically associated with conflict monitoring and cognitive control, reflecting the detection of conflicting stimuli and the engagement of inhibitory control mechanisms (Groom and Cragg, 2015; Kousaie and Phillips, 2017; Ji et al., 2023; Pfefferbaum et al., 1985; Sutton et al., 1965). The P3 component, on the other hand, is linked to the evaluation of stimuli and the updating of decision-making processes in response to task-relevant information (Donchin, 1981; Dong and Zhong, 2017; Kao et al., 2022; Rey-Mermet et al., 2019). However, for the head-fake task, which is more dynamic and involves continuous processing of both kinematic and deceptive cues, the ERP components to be analyzed were not predefined, and we adopted an exploratory approach. This analysis aimed to uncover how athletic expertise influences the neural mechanisms involved in processing sport-specific deceptive actions. Through this investigation, we aim to shed light on the role of athletic expertise in shaping both domain-specific and general cognitive control processes, particularly regarding information processing and conflict resolution.

## Methods

### Participants

A prior power analysis was conducted using G*Power (Faul et al., 2007) to estimate the required sample size for a 2 × 2 repeated measures ANOVA design. Based on the effect size *f* of 0.25, an alpha level of 0.05, a power level of 0.8, and assuming a correlation among repeated measures of 0.5, a sample size of 36 participants (18 per group) was deemed sufficient. Considering potential data loss due to EEG signal quality issues, we recruited a total of 48 male participants, including 24 collegiate basketball players and 24 control participants with no prior ball game training or expertise.

The inclusion criteria for basketball players required participants to hold at least a second-grade national player qualification, as per Chinese national standards, and to have undergone no less than 4 years of professional training with a school or higher-level team (i.e., training for more than 4 h per day, 5 days a week). Control participants had no formal basketball training or regular experience watching basketball matches. Four participants were excluded from the final analysis due to excessive artifacts or ocular movements. Consequently, the final analysis included 22 collegiate basketball players and 22 control participants (see Table 1 for detailed demographic information).

**Table 1:** Demographic information of participants.

	Experts	Controls
Number	22	22
Age, mean ± SD, years	19.32 ± 1.29	20.52 ± 1.99
Years of training, mean ± SD	6.86 ± 2.40	0
Training frequency (days/week)	5–7	0
Training time (hours/day)	4–5	0
Level achievement^a^	National Second Level, *n* = 12 National First Level, *n* = 10	/

a According to the General Administration of Sport of China, the ranking of athletic categories from lowest to highest is: National Second Level Athlete, National First Level Athlete, and National Elite Athlete.

All participants were right-handed, reported normal or corrected-to-normal vision, and had no history of psychological or neurological disorders. Participation was voluntary, and all participants provided informed written consent in accordance with the Declaration of Helsinki. The study was approved by the local ethics committee (No. 102772022RT069).

### Task and procedure

Participants were seated in a dimly lit, soundproof room, positioned approximately 70 cm from a 17-inch monitor with a spatial resolution of 1,024 × 768. They performed the head-fake, flanker, and face-viewpoint direction flanker tasks in randomized order to counterbalance potential practice and boredom effects. The total duration for completing all three tasks was approximately 40 min. Throughout the experiment, participants rested their left and right index fingers on the F and J keys, respectively. All tasks were programmed and administered using E-prime software (Psychology Software Tools, Inc., Pennsylvania, United States).

#### Head-fake task

Video clips were recorded depicting an elite basketball player dressed in black, standing in front of a white wall and passing a basketball. The recordings were captured using a digital video camera (Canon EOS 5D Mark IV; 60 Hz; resolution: 1,920 × 1,080 pixels). Each clip began with the player holding the ball centered in front of his body, looking directly at the camera. The player then passed the ball to either the left or right direction, with his head and legs turning either to the same or different direction. Previous research has primarily focused on the direction of the head as a task-irrelevant stimulus feature (e.g., Güldenpenning et al., 2022, 2023; Kunde et al., 2011; Weigelt et al., 2017). To eliminate the influence of leg direction, we ensured that the head and legs always turned in the same direction. A “head fake” was defined as the ball passing in a direction incongruent with the player’s head, while a “direct pass” referred to both the ball and head moving in the same direction. Based on these congruencies, four types of passes were used: head-fake and direct passes to both the left and right directions.

**Figure 1:**
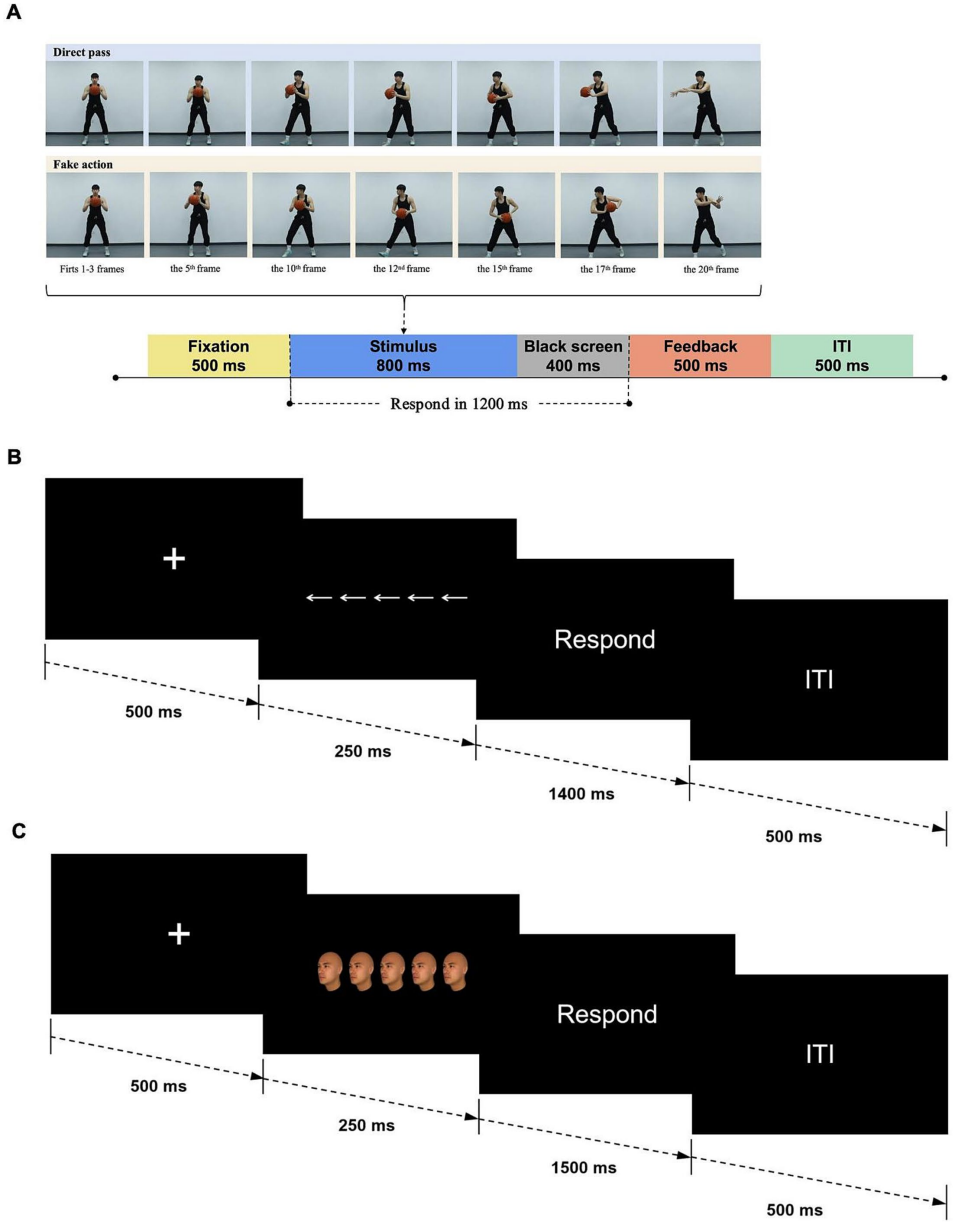
Schematic illustration of experimental trial structures and stimuli types. **(A)** Head-fake task. Photographs of a professional athlete were used in the schematic illustration. Written informed consent was obtained for the use of these images, with the participant fully aware of the study’s aims and the intended use of the images. **(B)** Flanker task. **(C)** Face-viewpoint direction flanker task.

For each type of pass, four video clips were selected, resulting in a total of 16 clips. These clips were processed using Adobe Premiere software (Adobe Systems Incorporated, San Jose, CA, United States), with each video clip consisting of 20 continuous frames (resolution: 800 × 700 pixels, 25 frames per second). Each clip began with the player standing still at the center of the frame (frames 1–3). From frames 4–16, the player executed the passing motion, and by frame 17, the ball had left his hands. The final three frames (18–20) showed the ball moving away from the player. Each frame lasted 40 ms, resulting in a total clip duration of 800 ms (Figure 1A). This setup was adapted from previous studies, with minor adjustments to ensure the duration from pass initiation to completion was identical across all stimuli (Güldenpenning et al., 2020, 2022, 2023). In each trial, a central fixation cross was displayed for 500 ms, followed by one of the video stimuli. After the clip (800 ms), a blank screen was shown for 400 ms. Participants were instructed to respond to the passing direction by pressing the corresponding key as quickly and accurately as possible (“F” for left and “J” for right). If an incorrect key was pressed or no response was made during the allotted time, feedback was provided on the screen for 500 ms: “错误” (incorrect) or “未反应” (no response). A fixed inter-trial interval of 500 ms followed before the next trial (see Figure 1A). Participants first completed 16 practice trials (one for each video stimulus) to familiarize themselves with the task. The formal experiment consisted of three blocks, each containing 80 trials, with short breaks between blocks as needed. The four type of passing video clip stimuli occurred in a random order with equal frequency, resulting in 120 congruent and 120 incongruent trials for each participant. The head-fake task took approximately 20 min, including instructions and practice trials.

#### Flanker task

In the flanker task (Eriksen and Eriksen, 1974), participants viewed rows of five arrows on the screen. The central arrow (target) pointed either left (“<”) or right (“>”), and was flanked by two arrows on each side (distractors) that either pointed in the same direction (congruent condition) or the opposite direction (incongruent condition). The arrows in each stimulus array were horizontally aligned in a row, with their horizontal positions symmetrically distributed relative to the central fixation point (0°). The central arrow was precisely positioned at 0° of visual angle. The flanking arrows were symmetrically arranged on either side, corresponding to −6.54°, −3.27°, 3.27°, and 6.54° of visual angle, respectively. Each arrow measured approximately 3.51 cm in width (104 pixels), subtending a visual angle of 2.87°. Based on the congruency between the central and surrounding arrows, four types of stimuli were used: congruent trials (all arrows pointing left or right) and incongruent trials (flanking arrows pointing in the opposite direction of the central arrow).

Each trial began with a central fixation cross displayed for 500 ms, followed by a stimulus consisting of five arrows for 250 ms. Participants were instructed to focus on the central arrow and press the “F” key if the central arrow pointed left, and the “J” key if it pointed right, as quickly and accurately as possible. After the arrows disappeared, a blank screen was shown for 1,400 ms. The next trial began following a fixed inter-trial interval of 500 ms (see Figure 1B). Participants first completed eight practice trials (two for each stimulus type) to familiarize themselves with the task. The formal experiment comprised three blocks of 80 trials each, with short breaks between blocks if needed. Congruent and incongruent trials were presented randomly and equally, resulting in a total of 120 congruent and 120 incongruent trials for each participant. The flanker task lasted approximately 10 min, including instructions and practice trials.

#### Face-viewpoint direction flanker task

Thirty-two neutral-expression face images were generated using FaceGen software (version 3.12, Singular Inversions Inc., Toronto, ON, Canada) to create young adult male faces with unique identities from various ethnic groups. The process involved generating random faces from “All Races” and adjusting the “Age” scale while keeping the “Caricature,” “Gender” (set to male), and “Asymmetry” scales at average and typical values. Each face was rendered at a viewpoint angle of 45–50° to the left or right, forming horizontally aligned rows of five identical faces. The central face served as the target, while the flanking faces acted as distractors. The faces in each stimulus array were horizontally aligned in a row, with their horizontal positions symmetrically distributed relative to the central fixation point (0°). The central face was precisely located at 0° of visual angle. The flanking faces were symmetrically arranged on either side, corresponding to −7.46°, −3.73°, 3.73°, and 7.46° of visual angle, respectively. Each face image measured approximately 5.11 cm × 3.61 cm (152 × 107 pixels), subtending 4.18° × 2.95° of visual angle. Based on the consistency between the direction of the central face and the flanking faces, four types of stimuli were used: congruent trials (all faces pointing left or right) and incongruent trials (central face pointing in the opposite direction to the flanking faces). This generated a total of 120 face arrays. The design of the stimulus materials was based on King et al. (2012), with minor adjustments made to meet the specific requirements of the current experiment.

As in the standard flanker task, each trial began with a fixation cross displayed in the center of the screen for 500 ms, followed by the face array for 250 ms, and a blank screen for 1,500 ms (see Figure 1C). Participants were instructed to respond as quickly and accurately as possible by pressing the “F” key with their left index finger if the central face was turned left, and the “J” key with their right index finger if the central face was turned right. The next trial began after a fixed inter-trial interval of 500 ms. The practice block consisted of 16 trials, ensuring participants were familiar with the task before proceeding to the formal experiment. The formal experiment included three blocks of 80 trials each, with short breaks between blocks if needed. Stimuli were presented in random order with equal frequency across the four conditions, and each image was repeated twice in each block, resulting in a total of 120 congruent and 120 incongruent trials per participant. The face-viewpoint direction flanker task took approximately 10 min, including instructions and practice trials.

### Behavioral data analysis

For each participant, accuracy and mean reaction time (RT) on correct trials were calculated separately for each of the three tasks (head-fake task, flanker task, and Face-viewpoint direction flanker task) under each congruency condition. RT was defined as the time between stimulus presentation and the key press. In the head-fake task, outlier RTs were identified using the median ± 1.5 × interquartile range (IQR) method, which is robust to skewed distributions and minimizes the impact of extreme values. In the flanker and face-viewpoint direction flanker tasks, where response times are relatively short, trials with an RT more than 1.5 IQR above the median or less than 100 ms were considered outliers and excluded from further analysis. The IQR method is effective for identifying extremely slow responses, but it is less sensitive to extremely fast, non-goal-directed responses. Therefore, a 100 ms threshold was used to ensure the exclusion of non-physiological responses or anticipatory button presses. For each task, the mean RT and accuracy were analyzed using separate mixed-design 2 (Group: expert vs. control) × 2 (Congruency: congruent vs. incongruent) ANOVAs. Group was treated as the between-subject factor, while Congruency was the within-subject factor.

### EEG recording and preprocessing

EEG signals were recorded using a 64-channel BrainVision system (Brain Products GmbH, Germany; pass band: 0.01–100 Hz; sampling rate: 1,000 Hz), with electrodes positioned according to the international 10–20 system. The FCz electrode served as the online reference, and AFz was used as the ground. To monitor eye movements and blinks, horizontal electrooculogram (HEOG) electrodes were placed at the outer canthi and vertical electrooculogram (VEOG) electrodes below the left eye. Electrode impedance was kept below 5 kΩ to maintain data quality throughout the recording.

EEG data were processed offline using the EEGLAB toolbox (Delorme and Makeig, 2004) and custom MATLAB scripts. Signals were re-referenced to the average of all electrodes, and finite impulse response (FIR) filters were applied to minimize phase distortion. First, a high-pass filter at 0.1 Hz was used to remove slow drifts, followed by a low-pass filter at 40 Hz to attenuate high-frequency noise. Both filters used a Hamming window for smooth transitions. A notch filter (48–52 Hz) was applied to suppress 50 Hz power line noise. Independent component analysis (ICA) was used to identify and remove artifacts such as eye blinks and muscle activity, with artifactual components identified based on their spatial and temporal patterns. To address concerns about potential distortions introduced by the high-pass filter (Acunzo et al., 2012), we also analyzed the ERP data without applying the high-pass filter. The results showed no meaningful differences in the overall patterns of ERP components across tasks and conditions. This confirms that the high-pass filtering did not affect the validity or reliability of our reported ERP findings.

The EEG signal was then downsampled to 500 Hz. For the head fake task, continuous EEG data were segmented into epochs from −200 to 800 ms relative to the onset of the video clip stimulus, with a baseline correction applied using the 200 ms pre-stimulus interval. For the flanker task and the face-viewpoint direction flanker task, data were epoched from −200 to 600 ms relative to the onset of the arrow or face array stimulus, with the same baseline correction applied. Epochs containing absolute voltages exceeding 100 μV at any electrode were rejected. Four participants were excluded from further analysis due to excessive artifact rejection (more than 50% of epochs) or technical issues resulting in data loss.

### ERP analysis

**Figure 2:**
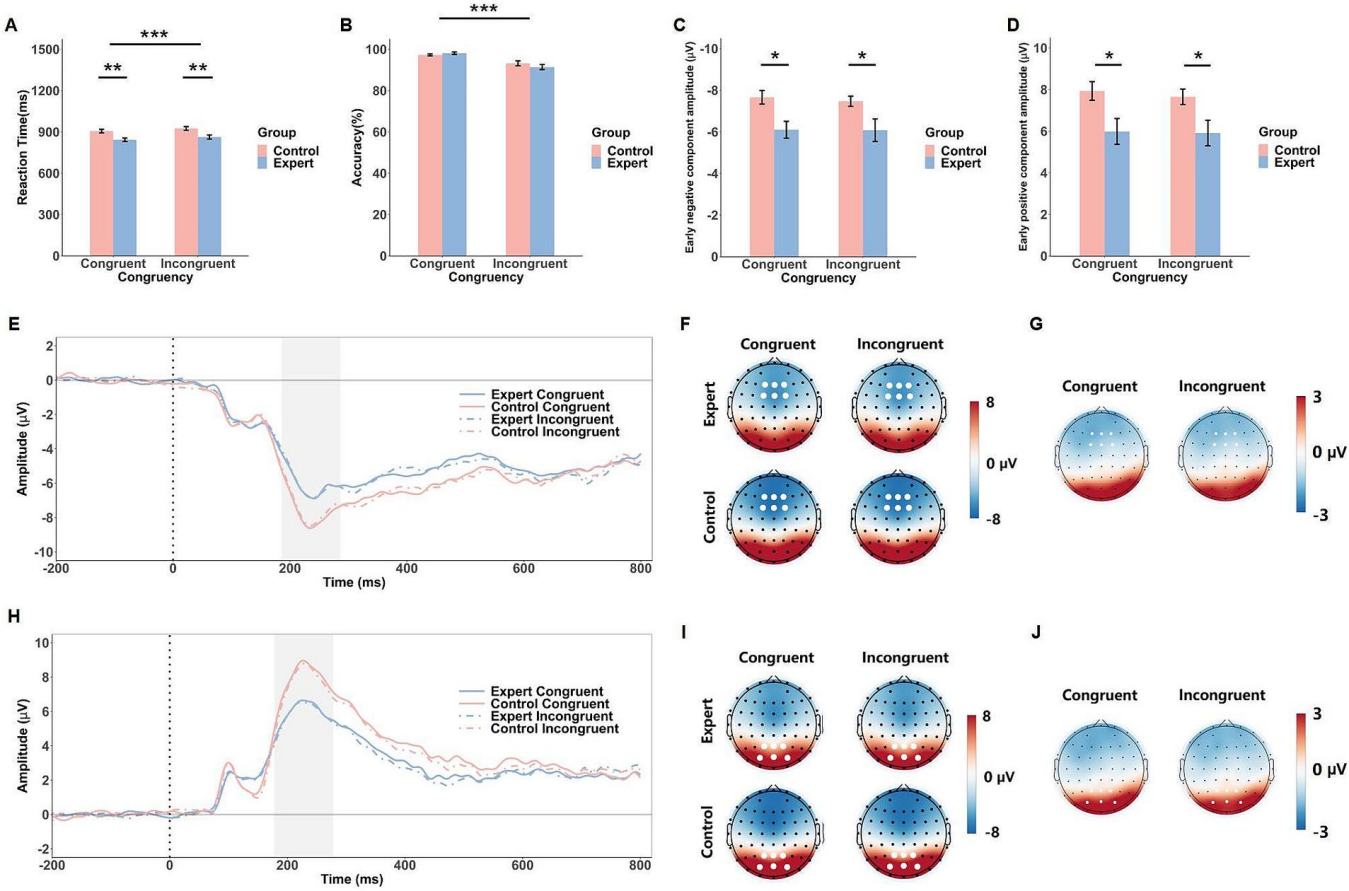
Behavioral and ERP results of the head-fake task. The mean RTs **(A)**, response accuracy **(B)**, early negative component amplitude **(C)** and early positive component amplitude **(D)** in response to the congruent and incongruent conditions in both groups (expert and control). Error bars show standard errors. Grand-averaged ERP waveforms **(E)** and corresponding scalp topographies within the 186–286 ms time window **(F)** for each congruency condition in both expert and control groups, with corresponding topographical maps of EEG differences between the expert and control groups **(G)**. Grand-averaged ERP waveforms **(H)** and scalp topographies within the 178–278 ms time window **(I)** are shown for each congruency condition in both expert and control groups, with corresponding topographical maps of EEG differences between the expert and control groups **(J)**. The gray shaded areas indicate the time windows used for ERP amplitude calculations. White dots represent the electrodes used to compute the ERP component amplitudes. Significance levels are denoted as ^*^p < 0.05, ^**^p < 0.01, and ^***^p < 0.001.

In the head-fake task, electrode selection was based on topographical maps to capture the regions with maximal activity for the components of interest. The components were analyzed at fronto-central electrodes (Fz, F1, F2, FCz, FC1, FC2; see Figure 2D) and parieto-occipital electrodes (Pz, P1, P2, POz, PO3, PO4; see Figure 2F). Due to the exploratory nature of this analysis, ERP components were not predefined. Instead, we identified an early negative component and an early positive component through visual inspection of the ERP waveforms. The time windows for these components were determined by identifying the peak amplitudes, with a ±50 ms window around the peak (e.g., 186–286 ms for the early negative component and 178–278 ms for the early positive component, as shown in Figures 2C,E).

For the flanker task, electrode and time window selection was based on previous literature (Hsieh et al., 2012; Tillman and Wiens, 2011; Wild-Wall et al., 2008). The N2 component was analyzed at frontal electrodes (Fz, F1, F2) in the 250–350 ms window following stimulus onset. The P3 component, related to stimulus evaluation and response selection, was measured at central electrodes (Cz, C1, C2) within the 350–500 ms time window.

In the face-viewpoint direction flanker task, the electrode sites and time windows were chosen to be consistent with those in the flanker task, as both tasks involve similar conflict and evaluative process. N2 was analyzed at frontal electrodes (Fz, F1, F2) between 250–350 ms post-stimulus, while P3 was analyzed at central electrodes (Cz, C1, C2) between 350–500 ms.

The mean amplitudes of the ERP components were calculated for each participant and condition across the time window and selected electrodes. Subsequently, the mean amplitudes were subjected to separate mixed-design 2 (Group: expert vs. control) × 2 (Congruency: congruent vs. incongruent) ANOVA, with Group as the between-subject factor and Congruency as the within-subject factor.

## Results

### Head-fake task

#### Behavioral results

The ANOVA on RT in the head-fake task revealed a significant main effect of Congruency, *F*(1, 42) = 23.11, *p* < 0.001, $\eta_{p}^{2}$ = 0.36, indicating that RTs were shorter in the congruent condition (M = 875 ms) than in the incongruent condition (M = 895 ms). There was also a significant main effect of Group, *F*(1, 42) = 10.92, *p* = 0.002, $\eta_{p}^{2}$ = 0.21, with shorter RTs observed in the expert group (M = 853 ms) compared to the control group (M = 916 ms). The Group × Congruency interaction was not significant, *F*(1, 42) = 0.01, *p* = 0.91 (see Figure 2A).

For accuracy, the ANOVA revealed a significant main effect of Congruency, *F*(1, 42) = 41.85, *p* < 0.001, $\eta_{p}^{2}$ = 0.50, showing that accuracy was higher in the congruent condition (M = 97.7%) than in the incongruent condition (M = 92.3%). However, neither the main effect of Group, *F*(1, 42) = 0.28, *p* = 0.63, nor the Group × Congruency interaction, *F*(1, 42) = 2.51, *p* = 0.12, reached significance (see Figure 2B).

#### ERP results

The ANOVA on the early negative component revealed a significant main effect of Group, *F*(1, 42) = 7.56, *p* = 0.009, $\eta_{p}^{2}$ = 0.15, with larger amplitudes in the control group (M = −7.57 μV) compared to the expert group (M = −6.09 μV). However, no significant main effect of Congruency, *F*(1, 42) = 0.45, *p* = 0.51, or Group × Congruency interaction, *F*(1, 42) = 0.29, *p* = 0.59, was found.

Similarly, for the early positive component, a significant main effect of Group was observed, *F*(1, 42) = 6.40, *p* = 0.015, $\eta_{p}^{2}$ = 0.13, with the control group showing larger amplitudes (M = 7.79 μV) than the expert group (M = 5.95 μV). No other significant effects were found (*F*s < 1.75, *p*s > 0.19, see Figure 2).

Further examination of the data suggests that the early negative and positive components observed in the head-fake task may represent opposite ends of a single dipole, as indicated by their highly similar morphology, peak latencies, and consistent pattern of amplitude differences between the groups.

### Flanker task

#### Behavioral results

**Figure 3:**
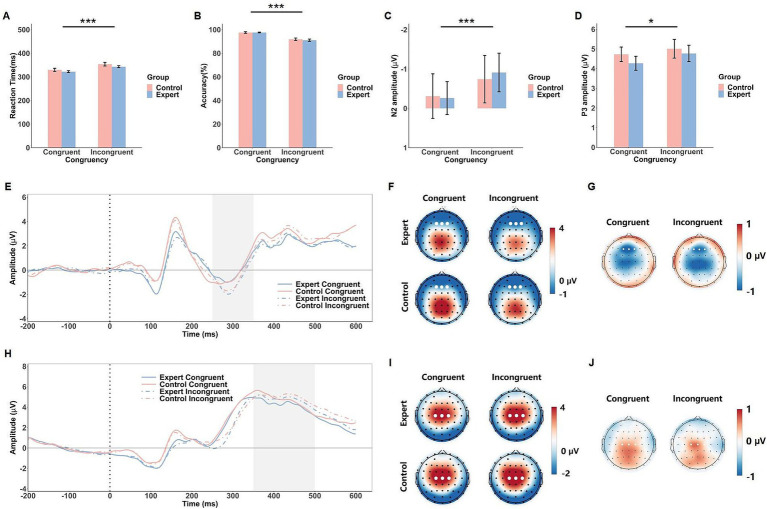
Behavioral and ERP results of the flanker task. The mean RTs **(A)**, response accuracy **(B)**, N2 amplitude **(C)** and P3 amplitude **(D)** in response to the congruent and incongruent conditions in both groups (expert and control). Error bars show standard errors. Grand-averaged ERP waveforms **(E)** and corresponding scalp topographies within the 250–350 ms time window **(F)** for each congruency condition in both expert and control groups, with corresponding topographical maps of EEG differences between the expert and control groups **(G)**. Grand-averaged ERP waveforms **(H)** and scalp topographies within the 350–500 ms time window **(I)** are shown for each congruency condition in both expert and control groups, with corresponding topographical maps of EEG differences between the expert and control groups **(J)**. The gray shaded areas indicate the time windows used for ERP amplitude calculations. White dots represent the electrodes used to compute the ERP component amplitudes. Significance levels are denoted as ^*^p < 0.05 and ^***^p < 0.001.

The ANOVA on RT in the flanker task revealed a significant main effect of Congruency, *F*(1, 42) = 122.67, *p* < 0.001, $\eta_{p}^{2}$ = 0.75, indicating that RTs were shorter in the congruent condition (M = 324 ms) compared to the incongruent condition (M = 346 ms). The main effect of Group, *F*(1, 42) = 2.45, *p* = 0.13, and the Group × Congruency interaction, *F*(1, 42) = 0.10, *p* = 0.75, did not approach significance (see Figure 3A).

For accuracy, the ANOVA revealed a significant main effect of Congruency, *F*(1, 42) = 95.85, *p* < 0.001, $\eta_{p}^{2}$ = 0.70, showing that accuracy was higher in the congruent condition (M = 97.6%) than in the incongruent condition (M = 91.5%). Neither the main effect of Group, *F*(1, 42) = 0.24, *p* = 0.63, nor the Group × Congruency interaction, *F*(1, 42) = 0.45, *p* = 0.51, reached significance (see Figure 3B).

#### ERP results

The ANOVA on the N2 component showed a significant main effect of Congruency, *F*(1, 42) = 15.95, *p* < 0.001, $\eta_{p}^{2}$ = 0.28, with larger N2 amplitudes in the incongruent condition (M = −0.83 μV) compared to the congruent condition (M = −0.29 μV). Neither the main effect of Group nor the Group × Congruency interaction reached significance (*F*s < 0.65, *p*s > 0.43). For the P3 component, there was a significant main effect of Congruency, *F*(1, 42) = 5.11, *p* = 0.029, $\eta_{p}^{2}$ = 0.11, with larger P3 amplitudes in the incongruent condition (M = 4.89 μV) compared to the congruent condition (M = 4.50 μV). No significant effects of Group or interaction were found (*F*s < 0.40, *p*s > 0.53, see Figure 3).

### Face-viewpoint direction flanker task

#### Behavioral results

**Figure 4:**
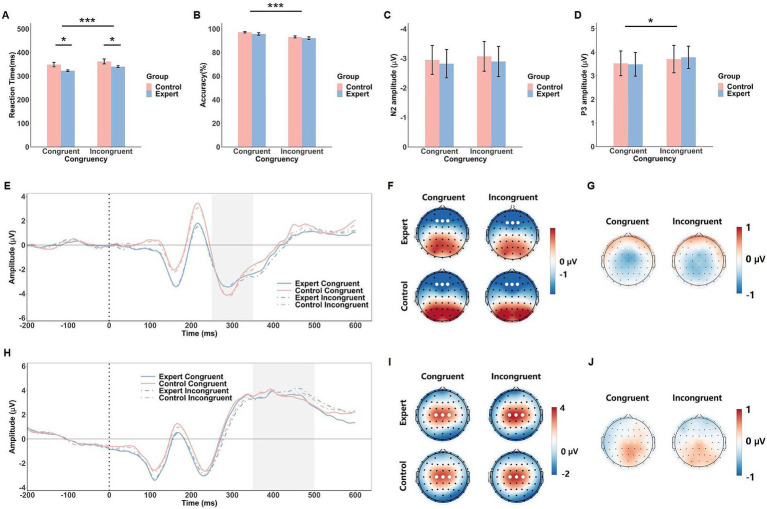
Behavioral and ERP results of the face-viewpoint direction flanker task. The mean RTs **(A)**, response accuracy **(B)**, N2 amplitude **(C)** and P3 amplitude **(D)** in response to the congruent and incongruent conditions in both groups (expert and control). Error bars show standard errors. Grand-averaged ERP waveforms **(E)** and corresponding scalp topographies within the 250–350 ms time window **(F)** for each congruency condition in both expert and control groups, with corresponding topographical maps of EEG differences between the expert and control groups **(G)**. Grand-averaged ERP waveforms **(H)** and scalp topographies within the 350–500 ms time window **(I)** are shown for each congruency condition in both expert and control groups, with corresponding topographical maps of EEG differences between the expert and control groups **(J)**. The gray shaded areas indicate the time windows used for ERP amplitude calculations. White dots represent the electrodes used to compute the ERP component amplitudes. Significance levels are denoted as ^*^p < 0.05 and ^***^p < 0.001.

The ANOVA on RT in the face-viewpoint direction flanker task revealed a significant main effect of Congruency, *F*(1, 42) = 162.13, *p* < 0.001, $\eta_{p}^{2}$ = 0.79, with participants responding more quickly in the congruent condition (M = 336 ms) compared to the incongruent condition (M = 351 ms). A significant Group effect was also found, *F*(1, 42) = 4.74, *p* = 0.035, $\eta_{p}^{2}$ = 0.10, indicating that the expert group (M = 331 ms) exhibited faster responses than the control group (M = 355 ms). The Group × Congruency interaction was not significant, *F*(1, 42) = 2.57, *p* = 0.12 (see Figure 4A).

For accuracy, the ANOVA revealed a significant effect of Congruency, *F*(1, 42) = 40.34, *p* < 0.001, $\eta_{p}^{2}$ = 0.49, where participants were more accurate in the congruent condition (M = 96.4%) than in the incongruent condition (M = 92.7%). In contrast, neither the main effect of Group, *F*(1, 42) = 1.12, *p* = 0.30, nor the Group × Congruency interaction, *F*(1, 42) = 0.12, *p* = 0.73, were statistically significant (see Figure 4B).

#### ERP results

The ANOVA on the N2 component did not reveal any significant main effects or interactions (*F*s < 0.30, *p*s > 0.58). However, for the P3 component, a significant main effect of Congruency was observed, *F*(1, 42) = 5.14, *p* = 0.029, $\eta_{p}^{2}$ = 0.11, with larger P3 amplitudes in the incongruent condition (M = 3.74 μV) compared to the congruent condition (M = 3.50 μV). No significant Group effect or interaction was found (*F*s < 0.80, *p*s > 0.38, see Figure 4).

## Discussion

Deceptive actions, such as head fakes in sports, create cognitive conflict by presenting irrelevant cues to mislead opponents, requiring individuals to resolve conflicting information (Güldenpenning et al., 2017; Jackson et al., 2006; Ripoll et al., 1995). To explore how athletic expertise shapes the processing of these deceptive cues and broader conflict scenarios, we examined behavioral and neural differences between basketball players and controls across three tasks: the head-fake task, which directly assesses sport-specific deceptive actions, and two general conflict control tasks—the flanker task and the face-viewpoint direction flanker task.

Athletes demonstrated shorter reaction times in both the head-fake and face-viewpoint direction flanker tasks than control participants, suggesting an expertise advantage, while no group differences were observed in the flanker task. All tasks showed a significant congruency effect, with participants responding more quickly and accurately in congruent conditions. ERP analyses revealed group differences in the head-fake task, with non-athletes exhibiting larger amplitudes for both the early negative and positive components. In the flanker task, both N2 and P3 were affected by congruency, whereas in the face-viewpoint direction flanker task, only P3 showed a congruency effect. No group differences were observed in either of the flanker tasks. Taken together, these findings suggest that athletes’ advantages are primarily related to their ability to process kinematic and social information, rather than superior conflict resolution abilities. While their expertise was evident in tasks involving social cues (e.g., head-fake, face-viewpoint direction flanker task), it did not extend to non-social stimuli, such as the arrows in the flanker task.

Behaviorally, the shorter reaction times exhibited by basketball players in the head-fake task indicate that athletes process kinematic information more efficiently compared to control participants. These findings align with prior research showing that skilled athletes excel at perceiving and interpreting complex motion cues, outperforming control participants (e.g., Aglioti et al., 2008; Lu et al., 2020; Zhao et al., 2018). Notably, basketball players also demonstrated shorter RTs in the face-viewpoint direction flanker task compared to control participants, emphasizing the advantage athletes have in processing social cues. One plausible explanation for this enhanced performance is that athletes, especially in sports like basketball, must continuously monitor both the physical actions and the facial cues of their opponents (e.g., gaze direction, viewpoint; Güldenpenning et al., 2017; Greenlees et al., 2005). In real game situations, interpreting facial cues is as critical as analyzing body movements to anticipate an opponent’s next action. This dual focus on kinematic and facial cues likely equips athletes with a significant advantage in extracting and interpreting relevant perceptual information during play. As such, the expertise effects observed in both the head-fake task and the face-viewpoint direction flanker task may reflect the result of extensive and repeated exposure to both motion-related and facial information during sports training (Broadbent et al., 2015; Raffan et al., 2024). However, given the cross-sectional design of this study, it is important to note that this approach does not allow for causal conclusions. Future research could investigate whether the observed advantages in processing these cues result from long-term training and the gradual development of expertise over time.

In contrast, our results from the flanker task revealed no significant group differences between athletes and non-athletes. Both groups performed similarly in terms of reaction time, suggesting that athletic expertise does not confer an advantage in processing non-social stimuli, such as arrows. This finding is crucial as it highlights that the expertise effect observed in the other two tasks is not due to a general faster response mechanism but rather to perceptual advantages specific to kinematic and social cue processing. If the shorter reaction times were solely due to general motor or response advantages, we would have expected athletes to outperform non-athletes in the flanker task as well. Additionally, prior research supports the distinction between social and non-social cue processing, indicating that different neural mechanisms are engaged when processing social cues like gaze or body movements versus non-social cues like arrows (Ji et al., 2020, 2022; Wang C.-H. et al., 2020; Wang L. et al., 2020; Yu et al., 2023). Body movements and physical actions can serve as social cues when they function as non-verbal signals conveying social information (Huang S. et al., 2024; Ji et al., 2020). In interactive contexts, these cues provide valuable insights into about emotional states or intentions and can be interpreted by others to guide social interactions and responses. Therefore, the behavioral results suggest that athletic expertise appears to enhance the processing of social, including kinematic cues, while non-social stimuli processing remains unaffected.

The congruency effects observed in all tasks indicate that participants—regardless of athletic expertise—consistently responded faster and more accurately in congruent conditions, a well-established effect in cognitive control tasks (e.g., Eriksen and Eriksen, 1974; King et al., 2012; Kunde et al., 2011; MacLeod, 1991; Simon, 1969; Stroop, 1935). This pattern is consistent with previous research on cognitive control and conflict monitoring, where congruent stimuli facilitate easier processing and reduce the need for cognitive control, resulting in faster and more efficient responses in the congruent condition compared to the incongruent condition (Botvinick et al., 2001; Egner et al., 2007). Importantly, no significant interaction effects between group and congruency were observed across the three tasks. This absence may reflect the nature of conflict resolution mechanisms, such as response inhibition and cognitive control, which may not be inherently enhanced by expertise alone. Prior studies employing tasks such as the flanker task have yielded limited and inconsistent evidence regarding the role of sports expertise in facilitating conflict resolution abilities. For example, Shao et al. (2020) examined shooting athletes using the flanker task and did not find a significant advantage in conflict resolution compared to non-athletes. Similarly, Krenn et al. (2018) investigated elite athletes from various sports and found no consistent evidence that sports expertise enhances conflict resolution abilities in the flanker task. These findings suggest that athletic expertise may not confer a generalized advantage in resolving cognitive conflict but rather refine specialized mechanisms for processing action-relevant visual information, as reflected in the behavioral results of the head-fake and face-viewpoint direction flanker tasks (Mann et al., 2007).

The ERP findings in the head-fake task revealed that non-athletes exhibited larger amplitudes for both the early negative and positive components compared to athletes, regardless of congruency, indicating distinct neural engagement between the two groups. Further analysis suggests that these two components observed in the head-fake task might reflect opposite ends of a single dipole, supported by the similar morphology and peak latencies observed across fronto-central and parieto-occipital electrodes. This suggests that these components could represent a common neural process, rather than being separate. Additionally, unlike the flanker tasks, where relevant and irrelevant information are presented simultaneously and transiently, the head-fake task presents video clips with relevant (pass direction) and irrelevant (head direction) information in a staggered and ongoing fashion. In the head-fake task, the irrelevant information (head movement) starts early in the clip and continues throughout, while the task-relevant information (pass direction) appears later. Specifically, the head movement begins around the 3rd frame (~120 ms), and the pass action starts around the 10th frame (~400 ms). In our selected time windows for the components, the congruent and incongruent information had not yet been presented. Given that the action sequence spans a longer duration and the components extend over a relatively long time window (as seen in the ERP waveforms in Figures 2E,H), it is plausible that these components reflect the processing of the entire action (including both head direction and pass direction) rather than solely resolving the conflict between the irrelevant and relevant information.

Regarding amplitude differences, athletes exhibited smaller ERP amplitudes compared to non-athletes, which, coupled with shorter reaction times, suggests that athletes may process kinematic information—such as body movements and pass direction—more efficiently. While the concept of neural efficiency posits that expert performers may engage fewer cognitive resources to achieve superior task performance (Del Percio et al., 2008, 2019; Li and Smith, 2021; Vickers and Williams, 2017), the observed amplitude differences in the head-fake task may indicate that athletes leverage their expertise in interpreting motion cues, allowing them to process this information with greater efficiency, potentially using fewer cortical resources and exhibiting smaller ERP amplitudes than non-athletes (Di Russo et al., 2005; Hatta et al., 2009; Li and Smith, 2021). However, it is important to consider that long-term training may lead athletes to adopt different processing strategies, involving distinct brain regions compared to non-athletes. This could result in a shift in dipole orientation that conflicts with the overall topography, which would not necessarily indicate neural efficiency, but rather a reorganization of brain activity. Thus, the interpretation of amplitude differences in the head-fake task remains speculative, and further research is required to provide clearer evidence.

In the flanker task, both N2 and P3 components showed significant congruency effects, with larger N2 and P3 amplitudes for incongruent trials. This pattern aligns with previous research, where incongruent stimuli heighten cognitive control demands, reflected in larger N2 amplitudes, while the additional cognitive workload required to resolve conflict often leads to enhanced P3 amplitudes as part of the evaluative and response-selection processes (Groom and Cragg, 2015; Kopp et al., 1996). The absence of group differences suggests that expertise related advantages are more specific to contexts involving kinematic and social information, rather than general cognitive processing. Additionally, within the selected time window, a brief period (~30 ms) was observed in the control group where the congruent condition exhibited a slightly higher amplitude than the incongruent condition. Given the short duration of this effect, it is likely a carryover effect from the preceding N2 component rather than a distinct P3 effect.

The face-viewpoint direction flanker task revealed a significant congruency effect for the P3 component, with larger amplitudes for incongruent trials, reflecting the increased cognitive workload required to process conflicting facial cues. This finding suggests that participants allocated more cognitive resources when resolving conflicts in facial cues, consistent with previous research on P3 as a marker of cognitive workload (Groom and Cragg, 2015; Kopp et al., 1996). However, while the N2 component was clearly present, it did not exhibit a significant congruency effect. One possible explanation is that the face-viewpoint direction flanker task, like the head-fake task, may involve an early negative component similar to what was observed in the head-fake task. Prior research suggests that social information engages distinct neural processes compared to abstract directional stimuli like arrows (Caruana et al., 2015; Huang Y. et al., 2024). The neural activity associated with early face perception might override or reduce the sensitivity of N2 to conflict detection in this context, diminishing its differentiation between congruent and incongruent conditions. Additionally, the relatively modest sample size in our study may have limited our ability to detect subtle neural differences, especially in components like N2, which exhibit considerable inter-individual variability (Leue et al., 2013; LoTemplio et al., 2021).

## Conclusion

In conclusion, this study demonstrates that athletic expertise enhances the processing of kinematic and social cues, though this advantage does not extend to non-social stimuli. The ERP results revealed that athletes exhibited smaller amplitudes in the early negative and positive components, which may reflect the processing of the entire action (including both head direction and pass direction) rather than solely resolving the conflict between irrelevant and relevant information. While all tasks exhibited a congruency effect, no group-by-congruency interactions were observed, suggesting that expertise does not provide a general advantage in conflict processing. Overall, these findings show that expertise specifically enhances the processing of kinematic and social information, rather than offering a broad advantage in managing cognitive conflict.

## Data Availability

The datasets presented in this study can be found in online repositories. Stimulus materials, raw data, and analysis scripts are available on the Open Science Framework (https://osf.io/6ftmx/).
